# Prevalence of Violence Perpetration by Men Aged 18–24 Years in Low- and Middle-Income Countries Who Were Exposed to Violence During Childhood — Eight Countries, 2018–2023

**DOI:** 10.15585/mmwr.mm7503a2

**Published:** 2026-01-22

**Authors:** Stephanie Spaid Miedema, Sarah A. Matthews, Francis B. Annor, Andrés Villaveces, Phumzile Mndzebele, Michelle R. Adler, Michelle Li, Kelly Ann Gordon Johnson, Denese McFarlane, Paul Rashad Young, Shelly Ann Edwards, Deidra Coy, Caroline Kambona, Elizabeth Washika, António Candeiro, Raquel Cossa de Pinho, Norbert Forster, Peter A. Minchella, Rahimisa Kamuingona, Laura F. Chiang

**Affiliations:** ^1^Division of Violence Prevention, National Center for Injury Prevention and Control, CDC; ^2^Division of Global HIV and Tuberculosis, Global Health Center, CDC Eswatini; ^3^Division of Global HIV and Tuberculosis, Global Health Center, CDC; ^4^Division of Global HIV and Tuberculosis, Global Health Center, CDC Jamaica; ^5^Planning Institute of Jamaica, government of Jamaica, Kingston, Jamaica; ^6^Division of Global HIV and Tuberculosis, Global Health Center, CDC Kenya; ^7^Division of Reproductive Maternal, Neonatal, Child and Adolescent Health, Ministry of Health, Republic of Kenya, Nairobi, Kenya; ^8^Division of Global HIV and Tuberculosis, Global Health Center, CDC Mozambique; ^9^Ministry of Health, Republic of Mozambique, Maputo, Mozambique; ^10^International Training and Education Center for Health, Windhoek, Namibia; ^11^Division of Global HIV and Tuberculosis, Global Health Center, CDC Namibia; ^12^Ministry of Gender Equality and Child Welfare, Republic of Namibia, Windhoek, Namibia.

**Summary**

**What is already known about this topic?**

Men aged 18–24 years (young men) disproportionately perpetrate many forms of interpersonal violence in low- and middle-income countries. Exposure to violence during childhood, including both experiencing and witnessing harm, might increase risk for perpetration of violence during adulthood.

**What is added by this report?**

In this survey study of young men in eight low- and middle-income countries during 2018–2023, lifetime prevalence of violence perpetration ranged from 12.4% in Eswatini to 44.9% in Côte d’Ivoire. Experiencing physical violence and witnessing violence in the household or community before age 18 years were associated with higher odds of perpetrating physical or sexual violence among young men in most countries.

**What are the implications for public health practice?**

Efforts to reduce exposure to violence during childhood might help reduce perpetration of violence among young men and make communities safer for all persons.

## Abstract

Violence is a major cause of morbidity and mortality among young adults in low- and middle-income countries. Men aged 18–24 years (young men) account for the majority of victims and perpetrators of many types of interpersonal violence. Childhood experiences, such as exposure to emotional, physical, or sexual violence or witnessing violence in their homes or communities, might increase risk for perpetration of violence in adulthood. Data from eight Violence Against Children and Youth Surveys conducted in low- and middle-income countries during 2018–2023 were analyzed to assess prevalence of physical and sexual violence perpetration by young men and associations of these events with their exposure to violence during childhood. Lifetime prevalence of physical or sexual violence perpetration among young men was common in all countries and ranged from 12.4% in Eswatini to 44.9% in Côte d’Ivoire. Physical violence victimization or witnessing violence in the household or community before age 18 years was associated with increased odds of violence perpetration among young men in all eight countries after adjusting for demographic covariates and childhood adversity indicators. Efforts to prevent exposure to violence during childhood, a pivotal developmental period, might reduce perpetration of violence by young men and create safer and more secure homes and communities.

## Introduction

Interpersonal violence is a leading cause of morbidity and mortality among adults aged 20–24 years (young adults) in many low- and middle-income countries (*1*,*2*). Young adults account for a higher incidence of most forms of interpersonal violence victimization and perpetration compared with adolescents (*1*). Although females also perpetrate violence (*3*), males are the primary perpetrators of interpersonal violence against both females and males (*1*,*4*). Childhood experiences, including exposure to violence, can increase the risk among boys for perpetration of violence when they are adults (*3*,*5*). Boys’ exposure to violence includes not only experiencing violence themselves but also witnessing violence in their household or community, places where they should feel safe and secure. Limited data are available for men aged 18–24 years (young men) in low- and middle-income countries (LMICs) regarding the association between witnessing violence during childhood and perpetration of physical or sexual violence during young adulthood (*3*). This nationally representative household survey study of young men in eight LMICs describes self-reported childhood emotional, physical, and sexual violence victimization and self-reported witnessing physical violence in the household or community, as well as the associations of these events with physical and sexual violence perpetration among young men. Findings can guide interventions to prevent violence victimization and perpetration among young men and promote safe and secure homes and communities.

## Methods

### Data Source

Since 2007, CDC, as a member of the Together for Girls (*6*) public-private partnership, has collaborated with more than 20 countries to implement the Violence Against Children and Youth Surveys (VACS). VACS are cross-sectional, nationally representative household surveys that assess emotional, physical, and sexual violence against persons aged 13–24 years (*7*). VACS uses a multistage cluster random sampling design. Primary sampling units are stratified by sex, and protocol protections are in place to secure the privacy and confidentiality of all respondents. During 2018–2023, CDC implemented VACS in collaboration with the governments of Colombia (2019), Côte d’Ivoire (2018), Eswatini (2022), Jamaica (2023), Kenya (2019), Moldova (2019), Mozambique (2019), and Namibia (2019). 

Standard VACS questionnaires were administered through face-to-face interviews with youths in all countries except Jamaica, which administered parts of the survey through audio computer-assisted self-interview software to comply with national mandatory reporting laws. Questionnaires were translated into the relevant local languages and back-translated to confirm accurate translation. Participation was voluntary and confidential. Informed consent was obtained. The survey included a tiered response plan that provided multiple levels of support intensity depending on participants’ exposure to adversities and need for social and health services. A general list of youth-friendly support services was provided to all participants. Design and sampling details for each country survey are available in VACS reports (*6*). This analysis includes data for men aged 18–24 years (Colombia, 674; Côte d’Ivoire, 617; Eswatini, 733; Jamaica, 353; Kenya, 408; Moldova, 412; Mozambique, 424; and Namibia, 565). Survey response rates among males were lowest in Moldova and highest in Côte d’Ivoire (Colombia, 47.5%; Côte d’Ivoire, 87.7%; Eswatini, 84.7%; Jamaica, 64.5%; Kenya, 66.5%; Moldova, 45.5%; Mozambique, 81.1%; and Namibia, 84.2%). Data from adolescent boys aged 13–17 years were excluded because the exposure period for childhood violence (i.e., occurring before age 18 years) was not complete. This activity was reviewed by CDC, deemed research not involving human subjects, and was conducted consistent with applicable federal law and CDC policy.*

### Case Definitions

**Lifetime history of violence perpetration. **Lifetime history of violence perpetration at the time of the survey was defined as ever having perpetrated physical or sexual violence toward a partner (i.e., a current or previous girlfriend, intimate partner, or wife) or nonpartner. Stratified multivariable analysis by partner versus nonpartner or physical versus sexual violence was not possible because of low prevalence of perpetration in some countries.

**Physical violence perpetration. **Physical violence perpetration was defined as ever slapping, pushing, shoving, shaking, or intentionally throwing something to hurt a person; punching, kicking, whipping someone or beating a person with an object; choking, smothering, or trying to drown or burn someone intentionally; or threatening someone with or using a knife, gun, or other weapon. Sexual violence perpetration was defined as forcing someone to have sex with the perpetrator when that person did not want to.

**Childhood physical and sexual violence victimization. **Childhood physical violence victimization was defined as ever having been slapped, pushed, shoved, or shaken; having had something intentionally thrown at them to hurt them; or having been punched, kicked, whipped, beaten with an object, choked, smothered, held under water, burned intentionally, or threatened with a weapon before age 18 years. Childhood sexual violence victimization included having experienced any unwanted sexual touching, attempted forced sex, physically forced sex, or pressured sex before age 18 years.

**Childhood emotional violence victimization. **Childhood emotional violence victimization before age 18 years was assessed using separate measures for three groups of perpetrators: 1) parent, adult caregiver, or other adult relative; 2) current or ex-intimate partner; or 3) peer. Emotional violence by a parent, adult caregiver, or other adult relative was defined as telling the participant they were not loved or did not deserve to be loved, saying they wished the participant had never been born or were dead, or ridiculing the participant (i.e., telling the participant that they were stupid or useless). Emotional violence by a current or ex-intimate partner was defined as insulting, humiliating or making fun of the participant in front of others; keeping the participant from having money; or trying to keep the participant from seeing or talking to friends or family. Emotional violence by peers was defined as making the participant feel scared or bad because of name-calling; saying mean things; saying they did not want the participant around; telling lies about, spreading rumors about, or trying to make others dislike the participant; keeping the participant out of things on purpose; excluding the participant from friend groups; or completely ignoring the participant.

**Witnessing violence during childhood.** Witnessing violence during childhood was assessed as the participant ever having seen or heard a mother or stepmother being hit, punched, kicked, or beaten by a father or stepfather; ever having seen or heard a parent punch, kick, or beat brothers or sisters; or ever having seen anyone being attacked outside of the home and family environment.

Demographic covariates were selected a priori based on evidence of confounding or moderating relationships with key indicators. Age was assessed as a continuous variable from 18–24 years. Schooling was assessed as whether participants attended the country-specific equivalent of primary school or less or of secondary school or higher. Marital status was assessed as whether youths had ever been married or cohabited. Current food insecurity was assessed as whether the participant indicated that the household did not have enough money for food. Exceptions were Kenya, where food insecurity was assessed as whether the participant went without food because there was not enough food in the household for ≥1 day during the past month, and Colombia, because data on food insecurity were not available.

### Data Analysis

Weighted percentages with associated 95% CIs were estimated for all variables. VACS weighting procedures enabled estimation of nationally representative data for each country. Multivariable logistic regression models were estimated separately by country to identify associations between childhood violence exposures (emotional, physical, sexual victimization, and witnessing violence) and violence perpetration, adjusting for demographic covariates (age, schooling, marital status, and food insecurity). All analyses included individual survey weights, accounted for VACS complex survey design, and were conducted using R software (version 4.5.0; R Foundation). For prevalence estimates, nonoverlapping 95% CIs were considered statistically significant. For multivariable models, p-values <0.05 were considered statistically significant. Missing data (missingness) for all variables was <3% in all countries except Côte d’Ivoire, Jamaica, and Namibia, where unweighted missingness ranged from 3.01% (ever witnessed violence during childhood [Namibia]) to 7.93% (ever perpetrated nonpartner sexual violence [Jamaica]). Missingness was assessed and taken into account using full information maximum likelihood estimation in all regression models.

## Results

Prevalence of having experienced and witnessed violence during childhood and of lifetime perpetration of violence varied across countries and by type of violence (Table 1). Physical violence was the most common form of childhood violence experienced by young men in most countries, ranging from 14.3% of participants in Eswatini to 60.8% in Côte d’Ivoire. Prevalence of childhood emotional violence victimization ranged from 5.0% in Kenya to 31.0% in Jamaica, and of childhood sexual violence victimization, from 2.1% in Eswatini to 11.7% in Jamaica. Witnessing violence during childhood was common: >60% of young men witnessed violence in their household or community before age 18 years in all countries except Eswatini (31.1%). Violence perpetration among young men was prevalent in all countries: prevalence of physical and sexual violence perpetration was highest in Côte d’Ivoire (44.9%) and lowest in Eswatini (12.4%). In all countries, perpetration of physical violence was more prevalent than that of sexual violence. Prevalence of nonpartner sexual violence perpetration was <3% in all countries. Prevalence of intimate partner sexual violence perpetration was highest in Côte d’Ivoire (8.5%) and Namibia (7.2%).

**TABLE 1:** Prevalence of childhood violence exposure and lifetime physical or sexual violence perpetration among men aged 18–24 in low- and middle-income countries — Violence Against Children and Youth Surveys, eight countries, 2018–2023

Variables*	Colombia 2019 n = 674		Côte d’Ivoire 2018 n = 617		Eswatini 2022 n = 733		Jamaica 2023 n = 353		Kenya 2019 n = 408		Moldova 2018 n = 412		Mozambique 2019 n = 424		Namibia 2019 n = 565	
	No.	% (95% CI)	No.	% (95% CI)	No.	% (95% CI)	No.	% (95% CI)	No.	% (95% CI)	No.	% (95% CI)	No.	% (95% CI)	No.	% (95% CI)
**Childhood violence exposure**																
Emotional violence victimization	87	9.5 (6.3–14.1)	84	15.5 (11.3–20.9)	78	9.9 (6.6–14.5)	95	31.0 (25.4–37.1)	20	5.0 (2.5–9.8)	40	8.9 (5.9–13.3)	30	6.2 (4.0–9.4)	47	7.8 (5.7–10.5)
Physical violence victimization	275	37.5 (28.5–47.5)	342	60.8 (54.0–67.2)	106	14.3 (10.6–19.0)	115	34.4 (28.4–41.0)	186	51.9 (43.2–60.5)	136	35.2 (27.7–43.5)	155	34.2 (27.2–41.9)	240	41.3 (34.8–48.0)
Sexual violence victimization	46	7.8 (4.8–12.5)	69	11.4 (7.7–16.5)	12	2.1 (1.1–4.0)	37	11.7 (7.9–17.1)	23	6.4 (3.6–11.3)	25	5.4 (3.3–8.6)	28	8.4 (6.2–11.4)	47	7.3 (4.9–10.9)
Witnessed violence during childhood	452	62.6 (53.7–70.8)	374	66.5 (61.5–71.1)	235	31.1 (25.6–37.1)	251	74.2 (68.9–78.8)	268	68.7 (61.8–74.9)	293	75.0 (68.1–80.8)	310	77.5 (71.3–82.6)	388	69.0 (60.8–76.2)
**Violence perpetration**																
Sexual nonpartner violence	—^†^	—^†^	14	1.8 (1.0–3.2)	—^†^	—^†^	—^†^	—^†^	—^†^	—^†^	—^†^	—^†^	16	3.0 (1.7–5.3)	—^†^	—^†^
Sexual intimate partner violence^§^	11	1.6 (0.6–4.1)	39	8.5 (5.9–12.2)	—^†^	—^†^	—^†^	—^†^	22	5.1 (2.9–8.7)	—^†^	—^†^	29	6.2 (4.1–9.3)	32	7.2 (4.0–12.6)
Nonpartner or intimate partner sexual violence	13	1.7 (0.7–3.9)	47	7.0 (4.9–10.0)	—^†^	—^†^	—^†^	—^†^	25	4.2 (2.4–7.4)	11	3.2 (1.2–7.9)	37	7.0 (4.9–10.0)	36	5.9 (3.5–9.9)
Physical nonpartner violence	119	23.3 (15.7–33.3)	167	29.2 (23.8–35.4)	94	11.2 (8.0–15.5)	55	16.1 (12.4–20.7)	37	19.1 (12.0–29.0)	40	9.3 (5.3–15.8)	52	10.9 (7.8–15.1)	95	16.1 (12.1–21.1)
Physical intimate partner violence^§^	42	5.6 (3.4–9.2)	103	26.7 (21.8–32.4)	18	2.1 (1.3–3.4)	28	8.5 (5.5–12.8)	50	16.5 (12.1–22.1)	—^†^	—^†^	58	12.6 (9.2–17.0)	50	9.3 (6.2–13.6)
Nonpartner or intimate partner physical violence	139	25.6 (17.6–35.6)	231	42.3 (36.8–47.9)	103	12.0 (8.8–16.2)	65	18.4 (14.4–23.2)	58	13.6 (10.3–17.7)	46	10.3 (6.1–17.0)	78	16.8 (13.7–20.4)	123	18.8 (14.7–23.6)
Physical or sexual violence	145	26.1 (18.1–36.1)	249	44.9 (39.7–50.3)	105	12.4 (9.1–16.7)	72	20.4 (16.5–24.8)	77	16.8 (13.3–21.1)	54	13.2 (8.6–19.7)	95	21.0 (17.4–25.1)	142	21.3 (17.2–26.1)
**Demographic covariates**																
Age, yrs (mean, SD)	20.8 (2.1)		20.6 (2.0)		20.8 (2.0)		20.5 (2.0)		20.5 (2.0)		20.5 (2.2)		20.8 (2.0)		20.5 (2.0)	
Ever married or cohabited	199	34.0 (25.9–43.1)	101	16.0 (12.1–20.7)	26	2.7 (1.7–4.3)	17	6.5 (3.8–11.0)	46	9.2 (6.9–12.1)	84	21.3 (15.9–27.9)	222	43.4 (36.1–51.1)	37	4.2 (2.8–6.4)
Attained secondary or higher education	627	94.5 (90.7–96.8)	378	63.5 (57.3–69.3)	639	87.5 (83.7–90.4)	334	96.6 (93.5–98.2)	294	74.4 (68.8–79.3)	361	90.6 (86.3–93.7)	160	44.2 (37.8–50.7)	462	81.5 (76.0–86.0)
Food insecure	NA	NA	300	48.7 (42.5–55.0)	453	62.7 (57.5–67.6)	80	24.5 (19.4–30.4)	103	26.3 (18.8–35.5)	40	8.4 (5.0–13.7)	269	58.5 (51.6–65.0)	308	63.1 (54.6–70.8)

**Abbreviation:** NA = not available.

* Missing data (missingness) on all variables was <3% in all countries except Côte d’Ivoire, Jamaica, and Namibia where unweighted missingness ranged from 3.01% (ever witnessed violence during childhood [Namibia]) to 7.93% (ever perpetrated nonpartner sexual violence [Jamaica]).

^† ^Estimate suppressed if n ≥10 and relative SE >30%.

^§^ Among adolescent boys and young men who had ever been married, cohabited, or dated (Colombia, 609; Côte d’Ivoire, 457; Eswatini, 564; Jamaica, 313; Kenya, 296; Moldova, 304; Mozambique, 395; and Namibia, 423).

After controlling for demographic characteristics and other forms of childhood violence victimization and witnessing violence in the home or community before age 18 years, the study found childhood physical violence victimization to be significantly associated with lifetime odds of physical or sexual violence perpetration in all countries except Mozambique (Table 2). After controlling for demographic characteristics and all forms of childhood violence victimization, the study found witnessing violence in the home or community before age 18 years to be significantly associated with lifetime odds of physical or sexual violence perpetration in five of the eight countries.

**TABLE 2:** Adjusted odds ratios of lifetime physical or sexual violence perpetration among men aged 18–24 in low- and middle-income countries — Violence Against Children and Youth Surveys, eight countries, 2018–2023

Variables*	aOR (95% CI)							
	Colombia 2019 n = 674	Côte d’Ivoire 2018 n = 617	Eswatini 2022 n = 733	Jamaica 2023 n = 353	Kenya 2019 n = 408	Moldova 2018 n = 412	Mozambique 2019 n = 424	Namibia 2019 n = 565
Age	1.0 (0.9–1.2)	1.1 (0.9–1.2)	1.2 (1.0–1.3)^†^	0.9 (0.7–1.1)	1.0 (0.8–1.2)	1.1 (0.9–1.3)	0.9 (0.8–1.1)	1.0 (0.9–1.2)
Secondary or higher education (ref: primary or less)	0.8 (0.3–2.8)	1.3 (0.8–2.2)	0.6 (0.3–1.3)	1.0 (0.4–3.1)	0.4 (0.2–0.9)	0.3 (0.1–0.9)	0.7 (0.3–1.4)	1.0 (0.5–1.8)
Ever married or cohabited	3.4 (1.3–8.9)^†^	2.4 (1.2–5.0)^†^	3.1 (0.9–10.2)	2.0 (0.5–9.2)	3.6 (1.3–10.4)^†^	2.2 (0.7–6.8)	1.7 (0.9–3.2)	2.7 (1.0–7.2)^†^
Food insecure	NA	1.2 (0.7–1.9)	0.7 (0.4–1.3)	0.8 (0.3–2.1)	0.3 (0.1–1.1)	1.0 (0.2–5.3)	0.5 (0.3–1.0)	1.4 (0.8–2.4)
Childhood emotional violence victimization	1.6 (0.7–4.1)	2.6 (1.5–4.6)^†^	2.1 (0.8–5.3)	1.1 (0.5–2.3)	6.0 (0.5–69.6)	0.6 (0.2–2.1)	2.8 (1.0–8.3)	0.8 (0.3–2.5)
Childhood physical violence victimization	4.8 (2.0–11.6)^†^	2.8 (1.5–5.3)^†^	10.9 (4.9–24.1)^†^	3.6 (1.8–7.4)^†^	2.8 (1.2–6.6)^†^	5.3 (2.4–11.7)^†^	2.0 (1.0–4.2)	3.2 (1.9–5.3)^†^
Childhood sexual violence victimization	0.8 (0.3–2.5)	1.3 (0.7–2.5)	0.2 (0.0–3.1)	3.1 (1.2–8.4)^†^	0.2 (0.0–1.4)	1.0 (0.2–4.2)	1.4 (0.5–3.6)	3.2 (1.5–6.9)^†^
Witnessed violence during childhood	5.1 (1.6–16.8)^†^	2.8 (1.6–4.8)^†^	2.7 (1.5–5.0)^†^	1.5 (0.7–3.0)	1.4 (0.4–4.8)	2.6 (0.7–9.6)	4.1 (1.6–10.9)^†^	4.2 (1.8–9.5)^†^

**Abbreviations**: aOR = adjusted odds ratio; NA = not available; ref = referent.

* Each country column represents a single multivariable regression model inclusive of all listed variables. Missing data (missingness) for all variables was <3% in all countries except Côte d’Ivoire, Jamaica, and Namibia, where unweighted missingness ranged from 3.01% (ever witnessed violence during childhood [Namibia]) to 7.93% (ever perpetrated nonpartner sexual violence [Jamaica]). Missingness was assessed and taken into account using full information maximum likelihood estimation in all regression models.

^†^ p<0.05.

## Discussion

Perpetration of physical or sexual violence among young men in the eight studied countries ranged from approximately 10% to approximately 40%. Results from this study corroborate findings from other low- and middle-income settings on men’s perpetration of violence (*5*) and suggest that when men do perpetrate violence, it can occur as early as adolescence and young adulthood (*1*). Witnessing violence in the household or community and experiencing physical violence before age 18 years were significantly associated with physical and sexual violence perpetration among young men in most countries. These results support global evidence on household violence in the lives of children (*8*) and childhood violence as a risk factor for men’s perpetration of violence in adulthood (*9*). Further, these results provide nationally representative data on the potential for household and community instability (e.g., witnessing violence in home and community spaces) to influence later harmful behaviors among young men. Efforts to prevent violence during childhood, a pivotal developmental period, and shift harmful attitudes and beliefs that normalize men’s use of violence can reduce perpetration of violence among young men and create safer and more secure homes, relationships, and communities (*10*). These efforts include implementation and enforcement of laws to prevent and respond to violence against children; shifts in restrictive or harmful social norms that support the use of violence; creation of safe physical and community environments for youths; improvement of family economic security and stability; improvements in access to health, social welfare, and justice services; and increases in children’s access to effective education and life-skills training (*10*).

### Limitations

The findings in this report are subject to at least six limitations. First, because VACS are cross-sectional, the temporality of indicators cannot be established. Second, because of the nature of data collected via VACS, establishing whether timing, severity, duration, or frequency of childhood violence victimization or exposure contributes to increased odds of violence perpetration is not possible. Third, data on food insecurity were not available in Colombia, and Colombia models were not adjusted for this possible confounder. Fourth, a skip pattern error in the Kenya VACS likely resulted in a downward bias of physical violence perpetration estimates in this country. However, estimates of physical violence perpetration from VACS data in neighboring Tanzania were not statistically significantly different from those in Kenya, suggesting that the bias might be relatively small. Fifth, violence data are self-reported and might be subject to reporting biases. Finally, because of sample size limitations, physical and sexual violence perpetration and partner and nonpartner perpetration were not disaggregated as separate outcomes.

### Implications for Public Health Practice

Understanding which risk factors are associated with violence perpetration among young men can help guide development of efficient violence prevention programs and services addressing population-specific factors to reduce future violence perpetration. Interventions to promote safe, stable, and nurturing childhood environments for male youths, such as school- and sports-based mentorship programs, might help to reduce the incidence of violence perpetration among young men (*10*). Structural efforts to improve community safety and promote youth community engagement and empowerment, such as after-school programming and safe transport options, could also contribute to reductions in violence perpetration and make communities safer for all youths (*10*). Prevention programs focused on male perpetration cessation might benefit from a trauma-guided approach that acknowledges the link between childhood exposure to violence and later perpetration of violence (*10*).

## Footnotes

* 45 C.F.R. part 46; 21 C.F.R. part 56; 42 U.S.C. Sect. 241(d), 5 U.S.C. Sect. 552a, 44 U.S.C. Sect. 3501 et seq.
